# Effects of a Sudden Drop in Salinity on *Scapharca subcrenata* Antioxidant Defenses and Metabolism Determined Using LC-MS Non-targeted Metabolomics

**DOI:** 10.1038/s41598-020-63293-0

**Published:** 2020-04-30

**Authors:** Mo Zhang, Li Li, Ying Liu, Xiaolong Gao

**Affiliations:** 1 State Key Laboratory of Marine Environmental Science, College of Ocean and Earth Sciences, Xiamen University, Xiamen, 361102 China; 2 Key Laboratory of Experimental Marine Biology, Institute of Oceanology, Chinese Academy of Sciences, Qingdao, 266071 China; 3 China Marine Biology Institute of Shandong Province, Qingdao, 266104 China; 4 Dalian Ocean University, Dalian, 116023 China

**Keywords:** Marine biology, Metabolism, Environmental impact

## Abstract

In this experiment, the effects of a sudden drop in salinity on the antioxidant defense system and related gene expression of the ark shell *Scapharca subcrenata* were examined. The sudden drop in seawater salinity after a rainstorm was simulated, and subsequently differentially expressed metabolic markers were identified by LC-MS non-targeted metabolomics. When the salinity dropped to 14‰ (S14), the total anti-oxidant content, activity of Na^+^/K^+^-ATPase, superoxide dismutase (SOD), and catalase (CAT), content of malondialdehyde, and expression levels of *Mn-SOD*, *CAT*, and C-type lectin of *S. subcrenata* were significantly higher than in groups with salinity of 22‰ (S22) or 30‰ (S30) (*P* < 0.05). The activity of glutathione peroxidase (GPx), the content of reduced glutathione, and the expression levels of *GP*_*x*_ were not significantly different between S14 and S22, but the values in each group were significantly higher than those in S30 (*P* < 0.05). Using the metabolomics technique, 361, 271, and 264 metabolites with significant differences were identified from S22 vs. S14, S30 vs. S14, and S30 vs. S22, respectively. The drop in salinity was accompanied by up-regulation of phosphatidylcholine (PC) (20:4 (5Z, 8Z, 11Z, 14Z)/P-18: 1 (11Z)), PC (16:0/22: 6 (4Z, 7Z, 10Z, 13Z, 16Z, 19Z)), phosphatidylethanolamine (PE) (18:4 (6Z, 9Z, 12Z, 15Z)/24:1 (15Z)), phosphatidylinositol (PI) (20:1 (11Z)/0:0), phalluside-1, C16 sphinganine, and LacCer (d18:0/14:0) and by significant down-regulation of PI-Cer (d18:1/14:0) and PE (14:0/16:1(9Z). The results of this study illustrate how these nine metabolites can be used as metabolic markers for the response of *S. subcrenata* to a sudden drop in salinity. They also provide the theoretical groundwork for selection of bottom areas with salinity that is optimal for release and proliferation of *S. subcrenata*, which is needed to restore the declining populations of this species.

## Introduction

The marine environment is being subjected to drastic changes due to global warming. Over the past 10–15 years, glaciers and ice sheets have disappeared at a fast rate, accompanied by heavy rainfall^1^. The large inflow of fresh water in different areas of the ocean has had various environmental impacts. The salinity of surface seawater and nearshore water is prone to a substantial decrease during the rainy season, resulting in massive mortality and changes in the distribution area of marine organisms^2^.

Salinity is a critical ecological factor in marine ecosystems that affects metabolism, osmotic adjustment, and the bio-rhythm of marine organisms^3,4^. Due to the effects of changes in the tide, surface evaporation, and seasonal rainfall, the salinity of seawater normally exhibits cyclic changes, and marine organisms have to adapt their own physiological activities to cope with such changes of salinity^5–7^. Marine shellfish are generally poikilosmotic animals (i.e., their internal and external osmotic pressures are equal). They can remain viable in salinities ranging from 4–5‰ to 75–80‰ depending on the species^8^. Under hypotonic conditions, poikilosmotic animals expand at a fast rate and slowly recover their normal body volume over time. However, the duration of recovery varies from species to species. Generally, organisms with a wider range of tolerance to salinity recover at a faster rate^9^.

Osmotic regulation by marine invertebrates is similar to that of vertebrates, and it includes perception, signal transduction, and physiological responses. However, the physiological response of invertebrates is quite different from that of homeosmotic animals. Firstly, to survive under unstable and volatile environmental conditions, most marine invertebrates have to reduce surface osmosis to a controllable extent in order to reduce the rate of change of ion concentration and water exchange. Next, the animal’s body processes Cl^−^ through the Na^+^ pump, although some tissues may process Cl^−^ through the anion pump. This ion exchange often occurs in the gills, excretory organs, epidermis, and other tissues and organs that have folded cell membranes and a large surface area^8^. The final step involves intracellular regulation. Under hypotonic conditions, when an organism has more dilute blood, cells retain ions and transport amino acids from the cell into the blood to reduce the osmotic gradient^10^. In this way, salinity can significantly affect various physiological functions of marine organisms. Therefore, determining how osmotic pressure is regulated is of great importance to understanding tolerance of marine shellfish to changes in salinity.

The ark shell *Scapharca subcrenata* is an economically important marine shellfish species in China. It typically is distributed in the subtidal zone between the low tide line and 7 m depth. Most individuals live in the fresh water-affected inner bay and neritic region. These animals show a wide range of adaptation to salinity and prefer to inhabit the soft mud or the muddy seafloor mixed with sand^11^. The environmental salinity range for *S. subcrenata* is approximately 17–37‰ with an optimum survivability between 23–33‰. The growth rate in lower salinity was faster than in high-salinity conditions. However, the survival rate was lower in low salinity compared to high-salinity conditions^12^. In *Anadara broughtonii*, the optimum conditions for growth, based on thean increase in shell length, was 26 °C with a salinity of 30‰^13^. Recently, overfishing and environmental changes have led to a drastic decrease in the availability of *S. subcrenata*. In order to restore the declining populations of *S. subcrenata*, large-scale enhancement and release of seeds have been conducted in some locations.

However, estuaries and intertidal zones are subject to drastic changes in salinity, which can negatively impact sessile or slow moving organisms. Such organisms can grow well within an appropriate range of salinity, but deviation from this range can retard growth of the population, lead to a stress response, and diminish immune resistance^14,15^. In the simulation that mimicked the sudden drop of salinity in seawater after a rainstorm, phagocytic activity of blood lymphocytes, O_2_^–^ levels produced from respiratory burst, and the activity of lysozyme and acid phosphatase were significantly higher in the 14‰ group compared to the 30‰ and 22‰ group. These results indicate that the immune defense mechanism of *S. subcrenata* was activated in response to salinity stress^16^. Therefore, to mediate the declining populations of *S. subcrenata*, a method is needed to identify optimal seed release areas based on the tolerance of this species to low salinity.

Metabolomics is an important component of systems biology, following genomics, transcriptomics, and proteomics. Metabolomics is a new analytical approach to identifying all of the low molecular weight metabolites in an organism or a cell. It is the most efficient method available for studying the relation of phenotype to function in an organism^17,18^. Although the experimental use of liquid chromatography-mass spectrometry (LC-MS) for metabolomics has been limited, it is suitable for the analysis of metabolites with poor volatilization or poor thermal stability. In addition, it offers high flux, resolution, and sensitivity. In this study, we used the LC-MS technique to evaluate the variation of metabolites and key metabolic pathways in the gills of *S. subcrenata* under different salinities that simulated the process of a sudden drop in seawater salinity after a rainstorm. Results of this study provide an understanding of the physiological adaptation process of marine invertebrates in response to low salinity.

## Materials and Methods

### Source and acclimation of *S. subcrenata*

*S. subcrenata* (shell length: 30.15 ± 1.46 mm, body weight: 7.97 ± 1.15 g) were purchased from Fuyuan Fisheries Company (Rizhao, Shandong, China), and all experimental *S. subcrenata* were sourced from the same batch after artificial hatching. After purchasing the ark shells, they were acclimated in culture containers (1.2 m × 1 m × 1 m, water volume: 1200 L) for 15 d; water temperature was kept at 20 °C, salinity at 29 + 1‰, pH at 7.9, dissolved oxygen concentration at >6 mg/L, and the light cycle was set as the natural light cycle. Aquaculture water was obtained from the natural sea area and used after sedimentation and sand filtration. Two-thirds of the water was replaced with fresh seawater each day at 09:00 to ensure good water quality. During the period of acclimation, the food mixture of *Chlorella vulgaris*, *Isochrysis galbana*, and *Platymonas subcordiformis* was provided at amount of 120 mL (once per day at a volume ratio of 1:1:1), and the food concentration was measured every 6 h.

### Experimental design

In this experiment, three salinity groups were established. Seventy-two individuals (three biological replicates × 8 *S. subcrenata* per biological replicate for each salinity group) of *S. subcrenata* that were cultured under normal seawater salinity of 30‰ were placed directly in seawater with salinities of 30‰ (S30), 22‰ (S22), or 14‰ (S14) for 72 h. The S30 group was used as the control. At the end of the experiment, six individuals of *S. subcrenata* were randomly selected from each group and shelled. Gill tissues were cut with scissors and removed with tweezers, then transferred to a 1.5 mL centrifuge tube. Excised tissue was immediately stored in liquid nitrogen for metabolomics research. Three additional individuals of *S. subcrenata* were randomly chosen from each group and shelled, and the gill tissues were cut, removed, rapidly placed in liquid nitrogen, and stored at −80 °C for later use in antioxidant enzyme assays and the related gene expression experiments.

### Determination of the activity of Na^+^/K^+^-ATPase and antioxidant-related enzymes in gill tissues

The activities of Na^+^/K^+^-ATPase, superoxide dismutase (SOD), catalase (CAT), glutathione peroxidase (GP_x_), total antioxidant capacity (T-AOC) and the contents of reduced glutathione (GSH) and malondialdehyde (MDA) were measured in gill tissue using kits purchased from Nanjing Jiancheng Bioengineering Institute (Nanjing, Jiangsu, China). Approximately 0.2–0.4 g of gill tissue were ground and solubilized in 1.8 mL of 0.86% saline in an ice-water bath. After grinding, samples were centrifuged at 3500 × *g*/min for 10 min until 10% tissue homogenates were obtained to measure enzyme activity.

Na^+^/K^+^- ATPase activity was measured as follows, 10% tissue homogenates were centrifuged at 4000 × *g*/min for 10 min, the supernatant was taken to prepare tissue homogenates with a volume fraction of 2%, and the enzyme activity was determined using the phosphorus determination method^19^. One unit of enzyme activity was defined as the generation of 1 μmol of inorganic phosphate from the decomposition of ATP per minute by ATPase in 1 mg of tissue protein^20^. The activity of SOD was determined using nitroblue tetrazolium (NBT) photochemical reduction: In the presence of methionine and riboflavin, NBT under illumination will generate blue methyl hydrazine through photochemical reduction^21^. The absorbance of blue methyl hydrazine was measured at 560 nm, and the enzyme activity was calculated by the intensity of SOD-inhibited NBT photochemical reduction. The activity of CAT was determined according to the method of Lygren *et al*.^22^. Briefly, 3 mL of 0.05 mol/L phosphate buffer was added to 200 μL of homogenate, followed by 200 μL of 0.3% H_2_O_2_. The reaction was mixed evenly. One minute later, the absorbance was measured at 240 nm, and it was recorded once every 1 min for 5 min. The DTNB (5,5′-Dithiobis-(2-nitrobenzoic acid)) method described by Gao *et al*.^23^ was used to measure GP_x_ activity. GP_x_ was assayed spectrophotometrically using glutathione as the substrate and the decrease of glutathione at 412 nm was measured.

T-AOC was determined using the ferric reducing ability of the plasma method^24^. The antioxidant substances in an organism can reduce Fe^3+^ to Fe^2+^, which can form a stable complex with phenanthroline-like substances. The level of T-AOC of a sample was determined using the colorimetric method. GSH content was measured following the method described by Anderson^25^, whereby one yellow compound can be generated when DTNB reacts with sulfhydryl compounds. The colorimetric method then was used to measure the absorbance of the yellow compound. MDA content was determined by measuring the amount of red product produced by MDA and tertiary butyl alcohol, which has a maximum absorption peak at 532 nm.

In these assays, absorbance was measured in nmol/mg protein using a spectrophotometer (721G-100, INESA.CC, Shanghai, China)^23^. The protein content in the homogenate was determined using Coomassie blue staining, as described by Bradford^26^, and bovine serum albumin was used as the protein marker.

### Extraction of metabolites

For the extraction of metabolites, the reaction mixture included approximately 30 mg of the sample, 20 μl each of the internal standard substances (L-2-chlorophenylalanine, 0.3 mg/ml; Lyso PC17:0, 0.01 mg/ml, prepared with methanol), and 400 μl of methanol (V_CH3OH_: V_H2O_ = 4:1). Two small steel balls were added, then pre-cooled at −20 °C for 2 min, and transferred to a grinder (60 Hz, 2 min). Tissue was extracted with sonication in a water bath for 10 min, followed by incubation in −20 °C for 20 min. Samples were then centrifuged for 10 min (13,000 × *g*, 4°C). After centrifugation, 200 μl of the supernatant was removed with a syringe, filtered through a 0.22 μm organic phase syringe filter, transferred to a LC vial, and stored at −80 °C until the LC-MS assay was conducted. Quality control (QC) samples were prepared by using extracts of all samples with equal volume, and the volume of each QC sample was the same as that of the experimental sample.

For the purpose of this experiment, the analytical instrument was an LC-MS system which consisted of an ACQUITY UPLC Ultra High Performance Liquid Chromatograph (Waters, Milford, MA, USA) in serial connection with an AB Triple TOF 5600 High Resolution Mass Spectrometer (AB Sciex, Redwood, CA, USA). The chromatographic column was an ACQUITY UPLC BEH C-18 (100 mm × 2.1 mm, 1.7 μm). The column temperature was 45 °C. The A and B mobile phases were water (with 0.1% formic acid) and B-acetonitrile (with 0.1% formic acid), respectively. The flow rate was set at 0.4 ml/min, and the injection volume was 5 μl. The ion source was electron spray ionization, and the sample MS signal was acquired using positive and negative ion scanning modes.

### Data pre-processing

Data were pre-processed prior to pattern recognition, and the raw data went through baseline filtering, peak identification, integration, retention time correction, peak alignment, and normalization using the metabolomics processing software, Progenesis QI v2.3 (Nonlinear Dynamics, Newcastle, UK). The main parameters were as follows: precursor tolerance: 5 ppm; product tolerance: 10 ppm; and product ion threshold: 5%. Compounds were identified based on exact mass number, secondary fragments, and isotopic distribution, and they were qualified using the Human Metabolome Database, Lipidmaps (v2.3), and the METLIN database. For extracted data, ion peaks with missing values (0 values) > 50% in the groups were deleted. The qualified compounds were screened according to the qualitative result scores of compounds. The screening standard was 30 points (full score: 60 points), and any compound with a score of <30 was considered to have inaccurate qualitative results and was deleted.

### Antioxidant-related gene expression of *Mn-SOD, CAT, GPx, CTL, HSP90*

Gill samples were removed from liquid nitrogen and then ground under liquid nitrogen with a mortar and pestle. Approximately 0.05 mg of the obtained sample powder was rapidly mixed with the 1 mL of TRIzol (Invitrogen, San Diego, CA, USA) to extract total RNA from the gills. Total RNA was extracted by removing the residual DNA from the sample using RQI RNase-Free DNase (TaKaRa, Kusatsu, Japan), and then RNA was reverse transcribed to cDNA using M-MLV reverse transcriptase (Promega, Madison, WI, USA). Real-time quantitative PCR was conducted using the SYBR^®^ Premix Ex Taq^TM^ II kit (Tli RNaseH Plus) (TaKaRa) and the TaKaRa Thermal Cycler Dice^TM^ Real Time System TP800 instrument. The specific primers were designed based on cDNA complete sequences submitted to GenBank, and *Mn-SOD*, *CAT*, *GP*_*x*_, C-type lectin (*CTL*), heat shock protein 90 (*HSP90*), and the reference gene β-actin were analyzed. Genetic information and primer sequences are presented in Table 1.

**Table 1:** Real-time quantitative PCR primers for antioxidant enzyme genes of *Scapharca subcrenata*.

Gene	Basic function	Sequence (5′-3′)	Size (bp)	Reference
Mn-SOD	Detoxification of O_2−_	F: CGGAACCACTCCTCCGTCA	167	Designed by author
		R: ACGCCGATTTTCTAACCGATT		
CAT	Detoxification of H_2_O_2_	F: GCCCACACCGAGACCTTAA	183	Designed by author
		R: AACTTAGCTAAGCGGGGTACC		
GP_x_	Detoxification of H_2_O_2_	F: ACTCCGCGCGTCCACG	149	Designed by author
		R: GCCATTTCACCTTTGGAT		
CTL	Detoxification of H_2_O_2_	F: CCTGGCTGACCCCAGTTCCCT	191	Shen *et al*.^68^
		R: ACCTGGGGAAGCCGGCAAAGT		
HSP90	Chaperone, Antioxidant biomarker of stress	F: GTAAAACCTCCAACAAAAGGCCCAGTT	134	Zheng *et al*.^69^
		R: CGAAAGCGCGGGCAAATCCGCAAGC		
β-Actin		F: ACACGGTAAAGCAACCTACC	207	Designed by author
		R: GCGCCCCAAACTTCCGAA		

Mn-SOD: Mn-superoxide dismutase; CAT: catalase; GP_x_: glutathione peroxidase; CTL: C-type lectin; HSP90: heat shock protein 90; F: forward primer; R: reverse primer.

Each sample was evenly mixed in a PCR tube and then placed in a PCR plate (Roche Diagnostics, Indianapolis, IN, USA). PCR amplification was conducted after transient centrifugation, and the reaction conditions were as follows: initial denaturation at 94 °C for 30 s followed by cycling at 94 °C for 5 s, 60 °C for 30 s for 40 cycles in total. The solubility curve was generated at the end of the experiment. Three replicates were run for each RNA sample and gene. mRNA levels of the target genes were calibrated using the Real-time PCR Ct (2^−ΔΔCt^) relative quantitative method, with β-actin as the quantitative standard.

### Data analysis

The data matrix was imported into the SIMCA software package (version 14.0, Umetrics, Umeå, Sweden). The overall distribution between samples and the stability throughout the analytic process were observed through unsupervised principal component analysis (PCA). The overall differences in metabolic profiles between groups were differentiated through orthogonal projections to latent structures discriminant analysis (OPLS-DA) in order to identify the metabolites that differed between groups. Differences in metabolites between groups were screened using multidimensional analysis in combination with unidimensional analysis. In the process of OPLS-DA, the variable influence on projection (VIP) analysis can be used to measure the influence and explanatory power of the expression pattern of each metabolite on the classification and discrimination of each group. Metabolites with VIP > 1 were considered to be differential metabolites. Further, a Student’s t-test was used to verify whether metabolite differences between groups were statistically significant. The screening criteria for the first principal component of the OPLS-DA model was VIP > 1, *P* < 0.05. Enrichment analysis of metabolic pathways was performed based on the Kyoto Encyclopedia of Genes and Genomes (KEGG) database (http://www.genome.jp/KEGG/pathway.html). Differential metabolites were mapped to the K EGG database to obtain the enrichment results of metabolic pathways. *P* < 0.05 was considered to indicate significant enrichment.

The statistical analysis was performed using SPSS, version 18.0 (IBM, Armonk, NY, USA). When one-way analysis of variance indicated statistical significance (*P* < 0.05), Tukey’s test was performed to examine the differences in antioxidant-related enzyme activity and gene expression of *S. subcrenata* in different salinity groups. Results are shown as the mean ± standard error. Sigmaplot (Systat Software Inc., San Jose, CA, USA) was used to draw the charts using the data obtained from the analyses.

### Ethics statement

All *S. subcrenata* in this study were handled in strict accordance with China’s legislation on scientific procedures on living animals. The protocol was approved by the ethics committee at University of Chinese Academy of Sciences (permit number: 399 20021109).

## Experimental Results

### Na^+^/K^+^-ATPase and antioxidant-related enzyme activity

The sudden drop in salinity had a significant effect on the activity of Na^+^/K^+^-ATPase and antioxidant-related enzyme activity (Table 2). The activity of Na^+^/K^+^-ATPase did not differ significantly between S22 and S30, but the value in both groups was significantly lower than that of S14 (*P* < 0.05). When the salinity decreased from 30‰ to 22‰, no significant difference in T-AOC was identified between the two groups. When the salinity decreased to 14‰, however, the T-AOC of *S. subcrenata* was significantly higher than that in the other two groups (*P* < 0.05). The SOD activity of *S. subcrenata* in S14 was significantly higher than that in any other group (*P* < 0.05). No significant difference in CAT activity and MDA content was identified between S30 and S22, but values in both groups were significantly lower than that in S14 (*P* < 0.05). No significant difference in GP_x_ activity and GSH content was identified between S30 and S22, but values in both groups were significantly lower than S14 (*P* < 0.05).

**Table 2:** Effects of sudden salinity changes on the total antioxidant capacity (T-AOC), contents of glutathione (GSH) and malondialdehyde (MDA), and activities of superoxide dismutase (SOD), catalase (CAT), and glutathione peroxidase (GP_x_) in *Scapharca subcrenata*.

Index	Salinity			ANOVA *P*
	30‰	22‰	14‰	
Na^+^/K^+^-ATP (μmol·Pi·mg^−1^ prot·h^−1^)	2.30 ± 0.74^b^	3.76 ± 0.82^b^	7.25 ± 0.56^a^	<0.001
T-AOC (U·mg^−1^ prot)	0.73 ± 0.02^b^	0.86 ± 0.03^b^	1.24 ± 0.02^a^	0.001
SOD (U·mg^−1^ prot)	12.18 ± 2.46^c^	19.33 ± 4.57^b^	28.05 ± 5.19^a^	<0.001
CAT (U·mg^−1^ prot)	9.52 ± 1.04^b^	15.79 ± 2.81^b^	37.59 ± 4.84^a^	<0.001
GP_x_ (U·mg^−1^ prot)	4.57 ± 0.86^b^	7.92 ± 0.63^b^	18.01 ± 3.23^a^	<0.001
GSH (mg·g^−1^ prot)	1.38 ± 0.08^b^	2.05 ± 0.37^b^	4.10 ± 0.56^a^	0.001
MDA (nmol·mg^−1^ prot)	3.05 ± 0.41^b^	4.97 ± 0.69^b^	11.75 ± 1.24^a^	<0.001

Values represent means and standard errors of three replicates. Values in the same row that have different superscripts are significantly different at *P* < 0.05 based on Tukey’s test. ANOVA: One-way analysis of variance.

### Base peak chromatograms (BPCs)

A BPC is a graph obtained by continuously plotting the ions with the highest intensity in the mass spectrum at each time point. Through the extraction and detection of *S. subcrenata* metabolites in S14, S22, and S30, positive ion BPC graphs of QC samples (prepared for the extracts of all samples with equal volume) contained 6659 original peaks. After the data went through standard pre-processing, 5995 peaks were retained. The negative ion BPC graphs contained 5128 original peaks, 4,527 of which were retained after data pre-processing (Supplementary Fig. 1).

### PCA analysis of all samples

**Figure 1:**
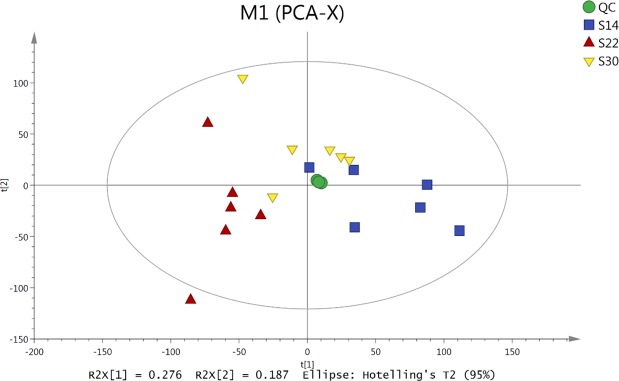
Score scatter plot of the principal component analysis (PCA) model for metabolites in *S. subcrenata* gills under different salinity conditions.

The PCA model graphs for *S. subcrenata* samples in each group were obtained through 7-fold cross-validation (Fig. 1). The PCA analysis showed that all samples were within the 95% confidence interval of Hotelling’s T2 test, suggesting that PCA can accurately describe the characteristics of the original data. In addition, using PCA it is possible to observe and predict the natural clustering trend of data between different salinity groups after dimension reduction. PCA shows an interesting pattern in which the S22 samples are clearly separated from the S14 samples on the first dimension, but S30 is more central and overlaps with the other two. QC samples were tightly clustered, indicating that the stability and repeatability of this experiment were credible and rigorous.

### Multivariate statistical analysis of two sample sets and response permutation tests (RPTs) of the OPLS-DA model

**Figure 2:**
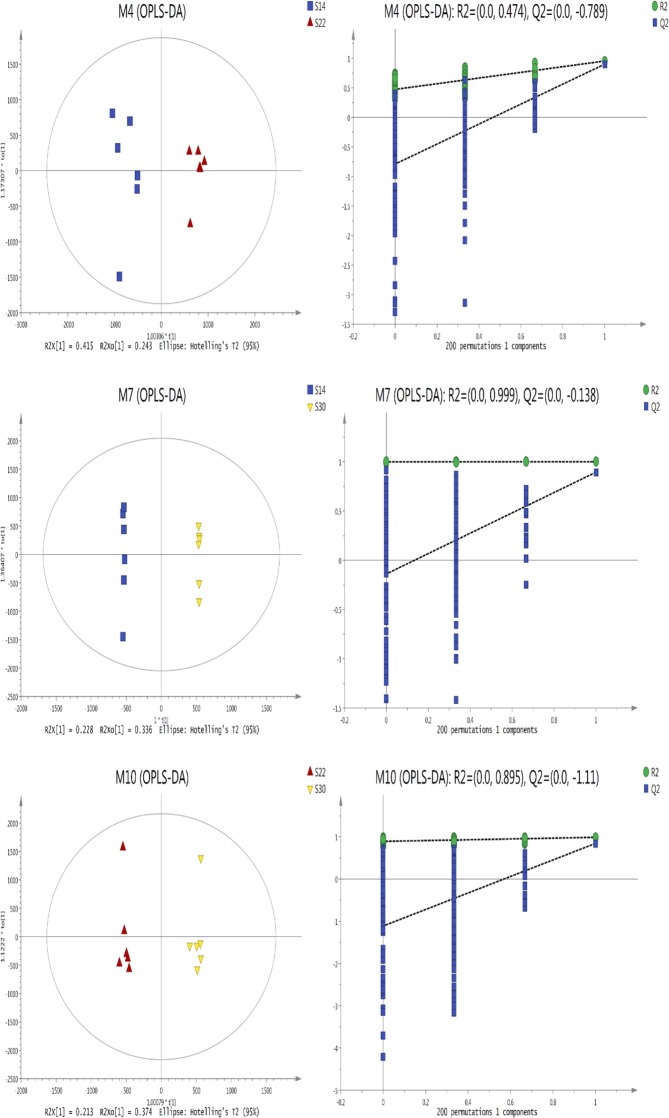
Score scatter plot and permutation test of OPLS-DA model for metabolites in *S. subcrenata* gills under S14 vs S22, S14 vs S30 and S22 vs S30.

Comparing the scatter plots and permutation test patterns for OPLS-DA scores pair-wise for the three salinity groups revealed that two treatment samples consisting of S14 and S22, S22 and S30, S14 and S30 were significantly differentiated in the predicted score for the first principal component (t[1]). Both samples were in the 95% confidence interval (Hotelling’s T2). Thus, the samples from different salinity levels could be differentiated based on the OPLS-DA analysis (Fig. 2).

To prevent the proposed model from overfitting, 200 RPTs were conducted on the OPLS-DA model. To be more specific, the X matrix was fixed and the predefined variables (such as 0 or 1) of the classification Y matrix were randomly permutated 200 times. The corresponding OPLS-DA model was established to obtain the R^2^ and Q^2^ values for the random model. Linear regression was performed with R^2^Y and Q^2^Y of the original model to obtain the intercept values of the regression line when the y-axes were R^2^ and Q^2^, respectively. The predictability of the proposed model for the differential metabolism between pair-wise comparison of S30, S22, and S14 [R^2^(Y)] was 95.40–100%. The cross-validity verification showed that the average predictability Q^2^Y was 84.60–90.10% and that Q^2^Y was >50%. This indicates that the model had good predictability. The permutation verification results from the OPLS-DA analysis showed that the regression line intercept, which was composed of the R^2^Y (as shown in the green circle) and the R^2^Y of the real model, was 0.474–0.999. In addition, the regression line intercept, which was composed of the Q^2^Y (as shown as blue diamonds), after 200 modeling trials and the Q^2^Y of the real model was −0.138 to −1.11. These results show that the established pattern recognition model was effective with no occurrence of overfitting.

### Identification and analysis of differential metabolites

**Figure 3:**
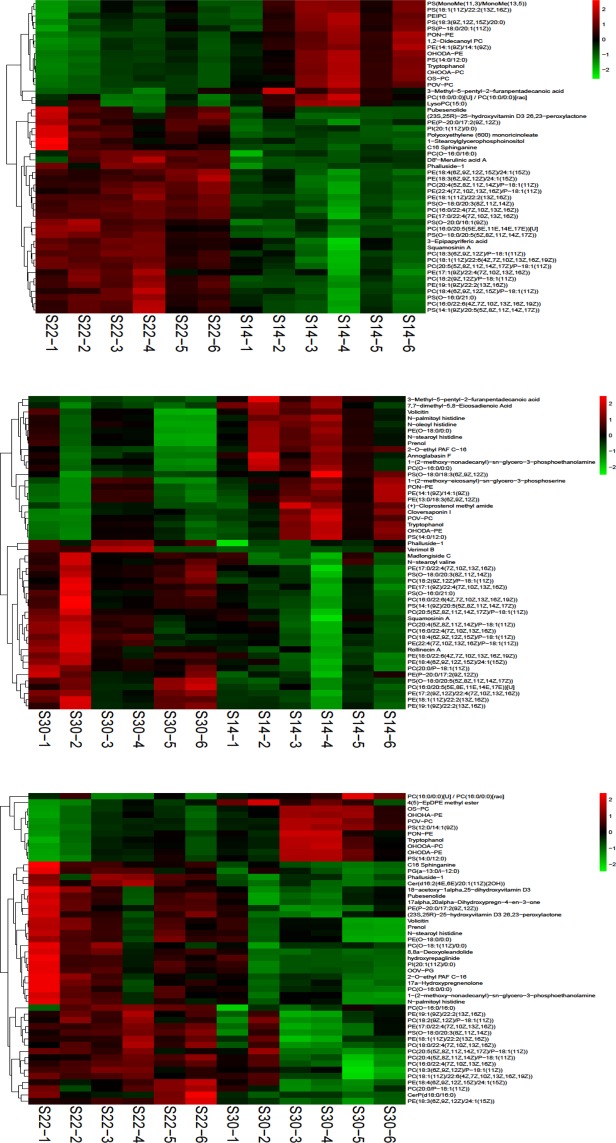
Hierarchical clustering analysis for the top fifty significantly different metabolites in S14 vs S22, S14 vs S30 and S22 vs S30. The relative metabolite level is depicted according to the color scale. Red indicates upregulation, and blue indicates downregulation.

In total, 361, 271, and 264 metabolites with significant differences were detected from S22 vs. S14, S30 vs. S14, and S30 vs. S22, respectively (VIP >1 and *P* < 0.05). The greatest number of differential metabolites were identified from S22 vs. S14 and included glycerophospholipids (phosphatidylcholine (PC) (20:4(5Z,8Z, 11Z,14Z)/P-18:1(11Z)), PC (16:0/22:6(4Z,7Z,10Z,13Z,16Z,19Z)), phosphatidylethanolamine (PE) (18:4(6Z,9Z,12Z,15Z)/24:1(15Z)), sphingolipids (phalluside-1, and C16 sphinganine.), fatty acyls (polyoxyethylene (600) monoricinoleate, 3-methyl-5-pentyl-2-furanpentadecanoic acid), steroids and steroid derivatives (pubesenolide), and sterol lipids ((23S, 25R)-25-hydroxyvitamin D3 26,23-peroxylactone). The differential metabolites identified from S30 vs. S14 also included glycerophospholipid (PC (20:4(5Z,8Z,11Z,14Z)/P-18:1(11Z)), PC (16:0/22:6(4Z,7Z,10Z,13Z,16Z,19Z)), PE (18:4(6Z,9Z,12Z,15Z)/24:1(15Z))), fatty acyls (N-stearoyl histidine, 3-methyl-5-pentyl-2-furanpentadecanoic acid), and indoles and indole derivatives (tryptophanol). The differential metabolites identified from S30 vs. S22 included glycerophospholipid (PC (20:4(5Z,8Z,11Z,14Z)/P-18:1(11Z)), phosphatidylinositol (PI) (20:1(11Z)/0:0), PE (18:4(6Z,9Z,12Z,15Z)/24:1(15Z)) *et al*.), sphingolipids (C16 sphinganine, phalluside-1, CerP(d18:0/16:0)), fatty acyls (N-stearoyl histidine, N-palmitoyl histidine), and steroids and steroid derivatives (pubesenolide) (Supplementary Table 1). To show the relationship between samples and the differential expression of metabolites between different samples, hierarchical clustering was performed using the expression levels of the top 50 metabolites that showed significant differences. The expression levels of Polyoxyethylene (600) monoricinoleate, 1-Stearoylglycerophosphoinositol, C16 Sphinganine, PC (O-16:0/16:0) increased in S22, but decreased in S14. The expression levels of PE (19:1(9Z)/22:2(13Z,16Z)), PC (18:2(9Z,12Z)/P-18:1(11Z)), and PE (17:0/22:4(7Z,10Z,13Z,16Z)) decreased in S30, but increased in S22. PC (18:4(6Z,9Z,12Z,15Z)/P − 18:1(11Z)), and PE (17: The expression level of 2 (9Z, 12Z)/22:4(7Z, 10Z, 13Z, 16Z)) decreased in S14, but increased in S30. The expression levels of differential metabolites were visualized based on VIP values (Fig. 3).

### KEGG annotation of differential metabolites

**Figure 4:**
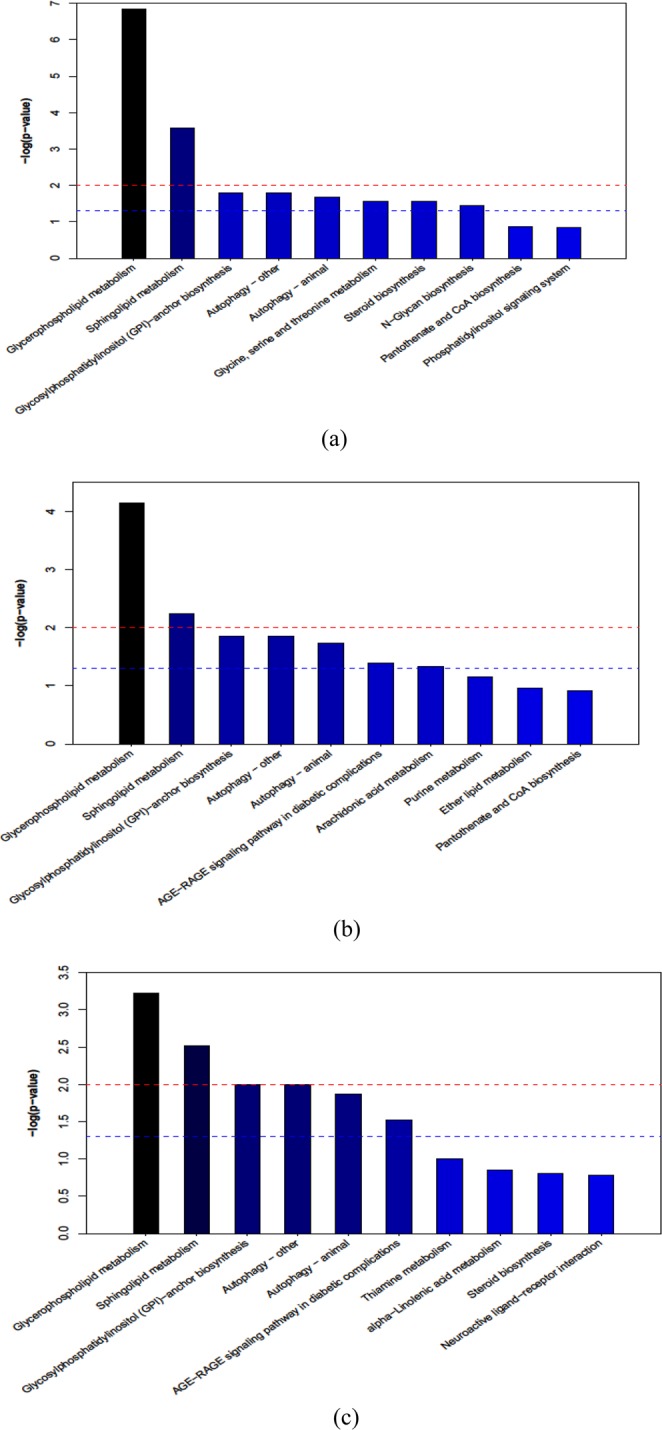
Metabolic pathway enrichment analysis in the S14 vs S22 (**a**), S14 vs S30 (**b**), and S22 vs S30 (**c**) treatment. The x axis represents pathway enrichment, and the y axis represents the pathway impact. Blue dashed line indicates *P* < 0.05, red dashed line indicates *P* < 0.01. The meaning of the changing colors of the bars is that the darker the color is, the more significant the difference in metabolic pathways would be.

Pathway enrichment analysis was performed using the KEGG ID of differential metabolites to derive the metabolic pathway enrichment results. There were 17, 16, and 13 metabolic pathways enriched from S22 vs. S14, S30 vs. S14, and S30 vs. S22, respectively. Of these, eight, seven, and six metabolic pathways were significantly enriched. Glycerophospholipid metabolism, sphingolipid metabolism, glycosylphosphatidylinositol (GPI)-anchor biosynthesis, autophagy-other, and autophagy-animal were significantly enriched in all three of the comparison groups (Fig. 4).

### Expression of antioxidant-related genes

**Figure 5:**
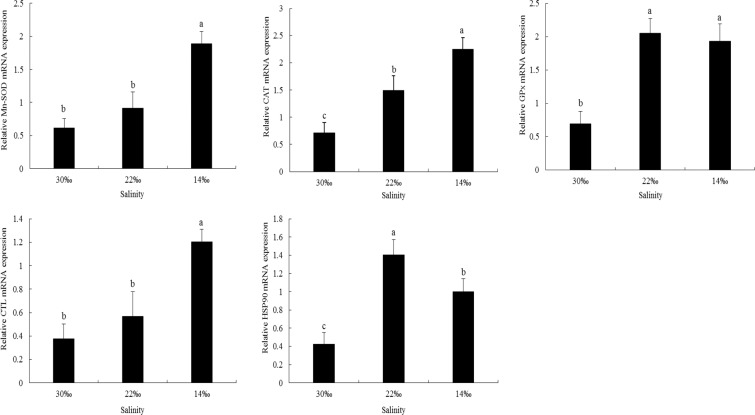
Relative mRNA expression levels of antioxidant enzyme and heat shock proteins’ genes in the gills of *Scapharca subcrenata*. Values are expressed as mean ± SE (n = 3). Statistical analysis was performed by one-way analysis of variance (ANOVA) followed by Tukey’s test, using SPSS version 18.0. Means with different lower case letters are significantly different at *P* < 0.05 level.

The expression levels of *Mn-SOD* gradually increased as the salinity decreased (Fig. 5). No significant difference in the expression levels of *Mn-SOD* was identified between S30 and S22, but the values in both groups were significantly lower than that in S14 (*P* < 0.05). Overall, the expression levels of *CAT* and *GP*_*x*_ in S14 and S22 were significantly higher than those in S30 (*P* < 0.05). No significant difference in the expression level of *CTL* was detected between S22 and S30, but the expression level of *CTL* was significantly higher in S14 (*P* < 0.05). As the salinity decreased, the expression level of *HSP90* tended to rise and then fall. The expression level of *HSP90* in S22 was significantly higher than that in any other group, and the value in S14 was significantly higher than that in S30 (Fig. 5, *P* < 0.05).

## Discussion

### Effects of a sudden drop in salinity on the activity of Na^+^/K^+^-ATPase

Salinity is a factor that limits the survival and distribution of many marine organisms. Specifically, a decrease in salinity can lead to massive mortality and migration of some species. Most marine invertebrates are poikilosmotic animals, in which the osmotic levels in the blood are close to that of seawater^27^. In response to environmental salinity changes, aquatic organisms passively lose or absorb water, causing changes in osmotic pressure. This promotes changes in metabolism, enabling Na^+^/K^+^-ATPase to be activated just until the osmotic pressure reaches equilibrium. Thus, the activity of Na^+^/K^+^-ATPase is significantly correlated with regulating osmotic pressure^28,29^. In the current study, the sudden drop in salinity was accompanied by a gradual increase in the activity of Na^+^/K^+^-ATPase, and the value in S14 was significantly higher than that in S30 or S22. This variation in the activity of the Na^+^/K^+^-ATPase indicates that when external osmotic pressure is decreased, the ions in the hemolymph tend to efflux. At this point, the activated Na^+^/K ^+^-ATPase not only supplied Na^+^ to the hemolymph to maintain the balance of ions, but it also provided a stable ion gradient to replenish other positive cations to cells so that the polarity difference of ions formed in and out of the cells was effectively alleviated. As a result, an effective alternative to maintaining the internal and external balance of water and salt was initiated, thus improving the organism’s adaptability to low salinity.

### Effects of a sudden drop in salinity on the antioxidant system of *S. subcrenata*

Non-enzymatic and enzymatic systems determine the T-AOC, and the enzymatic system includes SOD and GP_X_. The antioxidant system removes excess reactive oxygen species (ROS) to protect tissues from oxidative damage^30^. As the salinity decreased in this study, T-AOC gradually increased, and the value in S14 was significantly higher than that in S30 or S22. The cells in living organisms are often subject to oxidative stress as a result of internal metabolic changes in adjacent cells or tissues^31,32^, and the presence of antioxidant enzymes, antioxidant functional proteins, and GSH in the cells contributes to internal cellular homeostasis^33^.

SOD is a critical antioxidant enzyme in the body. It can remove O_2−_ free radicals, converting O_2−_ into H_2_O_2_, which then is decomposed by CAT and glutathione S-transferase (GST) so that free radicals can be maintained at a lower state of equilibrium to prevent oxidative damage to functional macromolecules^34,35^. The activity of SOD increased significantly from S30 to S22 to S14, which indicates that a sudden drop in salinity resulted in a large amount of ROS in the body. The enhanced activity of SOD is the key to accelerating the conversion of O_2−_ to H_2_O_2_.

Transcriptomics analysis (No. PRJNA548266) shows that *CTL* played an important role in immune regulation *S. subcrenata* as a response to sudden changes in salinity^16^. The expression levels of *CTL* and the activity of CAT were not significantly different between S30 and S22, which might be related to the wide tolerance of *S. subcrenata* to salinity. However, when the salinity decreased to 14%, the activity of CAT and the expression levels of *Mn-SOD*, *CAT*, and *CTL* significantly increased and the expression level of *GP*_*x*_ decreased. These results indicate that CAT and the GSH produced by the prior-stage catalysis of GP_x_ were enough to maintain the balance of the oxidation and reduction states. The kinetics of GP_x_ in mammals and other vertebrates shows that this enzyme has a strong affinity for H_2_O_2_^36,37^. In other words, it is GP_x_ in vertebrates that completes the removal of H_2_O_2_. However, CAT and GP_x_ can complement each other when scavenging H_2_O_2_^38,39^. This explains why the expression level of *GP*_*x*_ in S14 were not significantly different from that in S22.

MDA is the end decomposition product of lipid peroxidation, and its content levels reflect the degree of oxidative damage to the cell membrane. Therefore, it is extensively used as an indicator of oxidative damage to the plasma membrane^40^. In S14, the content of MDA was significantly higher than that in any other group, indicating that the cell membrane may be vulnerable to oxidative damage. However, a significant increase in the activity of CAT and the expression levels of CAT would accelerate the process of scavenging the free radicals produced from catabolism, thereby preventing damage to cell structure and function.

HSPs have been widely studied as biomarkers, as they not only respond to stress as a result of temperature change or hypoxia, but they also are triggered by exposure to heavy metals or free radicals. The heat shock stress response upregulates gene expression and subsequent protein levels in the cell^41,42^. With the sudden drop in salinity, the expression levels of *HSP90* did not rise continuously but instead increased from S30 to S22, then decreased in S14. We presumed that 14‰ salinity exceeded the tolerance limit of *S. subcrenata*, and cell function may have been inhibited by the oxidative stress. Wu *et al*.^43^ reported that the content of HSP90 mRNA stored in the cells for the maintenance of normal physiological function will be upregulated under early stages of environmental stress. It has also been shown that cysteine residues, methionine residues, and others in HSP90 are vulnerable to oxidation. However, given that the cysteine, methionine, and other residues in HSP90 are vulnerable to oxidation, long-term environmental stress may reduce the ability of HSP90 to act as a molecular chaperone. This phenomenon may explain why the *HSP90* expression levels first increased and then decreased. In a low-salinity state, the transcription level expression of antioxidant genes (e.g., SOD, copper-zinc SOD, CAT) in gill tissues of the abalone *Haliotis discus* significantly increased, as well as expression of immune response genes^44^. Gao *et al*.^45^ simulated the falling then rising trend of seawater salinity after a rainstorm and found that the expression levels of *CAT*, *TP*_*x*_, *GST*_*s*_, and *GST*_*m*_ tended to rise then fall, and the values were significantly higher than those in groups with constant salinity. As a result, under environmental stress, increased expression levels of antioxidant genes in the body might function as a tissue-specific protective mechanism and a long-term cytoprotective mechanism, which is of great significance for improving resistance to oxidative stress.

### Effects of a sudden drop in salinity on the lipid metabolism of *S. subcrenata*

Lipid metabolism is a critical component of metabolomics research. Lipids are the basic organic macromolecule of biomembranes as well as a potential energy source. In addition, lipids function as important signaling molecules and are essential in many biochemical pathways in the body. Currently, lipids such as glycerophospholipids, sphingolipids, glycerolipids, fatty acids, sterol lipids, saccharolipids, prenol lipids, and polyketides are classified in various organisms according to their structure and function^46^. In the present study, the signaling pathway of glycerophospholipid metabolism was significantly enriched in each salinity group. In S22 vs. S14, S30 vs. S14, and S22 vs. S30, PC (20:4 (5Z, 8Z, 11Z, 14Z)/P-18:1(11Z)), PC (16:0/22:6(4Z, 7Z, 10Z, 13Z, 16Z, 19Z)), PE (18:4 (6Z, 9Z, 12Z, 15Z)/24:1 (15Z)), and PI (20:1(11Z)/0:0) were up-regulated.

Glycerophospholipids are important carriers of fatty acids, and they help maintain the basic structure and morphology of cells. In addition, they serve in the recognition and uptake of cellular signaling substances and signal transduction^47^. Both PC and PE serve as critical components in the biological membrane. In addition, they help maintain normal cellular homeostasis and metabolism of the human body^48^. Given a decrease in salinity, active transport of inorganic ions (e.g.: K^+^, Na^+^, Cl^−^), which are the main osmotic effectors in the hemolymph of marine shellfish, often occurs during osmotic pressure regulation^49^. In this process, up-regulation of PC and PE is an effective means of maintaining the stability of the cell membrane structure and reducing the fluidity of the cell membrane, which in turn effectively enhance adaptation of the body to the sudden drop in salinity. The gonad lipids of the sea urchins *Hemicentrous pulcherrimus* and *Strongylocentrotus nudus* can effectively scavenge DPPH (1,1-diphenyl-2-picrylhydrazyl) free radicals, hydroxyl radicals, and superoxide anion radicals, and the scavenging capacity increases as the radical concentration increases in a dose-dependent manner^50^. In the present study, the sudden drop in salinity was thought to cause oxidative damage to the body, and up-regulation of glycerophospholipids may have provided a potent antioxidation effect, thus protecting important biomembranes (e.g., cell membrane, mitochondrial membrane) from being damaged. This upregulation may also have the added benefit of protecting macromolecules such as nucleic acids and proteins^51^.

Sphingolipid is a complex compound with sphingosine as the backbone that includes sphingomyelin, glycosphingolipid, and ceramide. It functions in maintaining the basic structure of cells and the basic activities including cell adhesion, growth, differentiation, proliferation and signal transduction^52,53^. In the current study, the sphingolipid metabolism signaling pathway was not only significantly enriched in each salinity group, but sphingolipid metabolites, including phalluside-1, C16 sphinganine, and LacCer (d18:0/14:0), also were increased in abundance in each salinity group. Conversely, PI-Cer (d18:1/14:0) was decreased in abundance. A change in membrane lipid composition may result in alteration of membrane fluidity and deformability, which in turn would affect the normal function of the cell membrane. Under a sudden drop in salinity, when ions frequently move in and out of the cell membrane, maintaining the integrity of the cell membrane and improving its permeability are important for enhancing the environmental adaptability of the body. The sphingolipids of all vertebrate nuclei play an important role in signaling and regulation. The sphingomyelin in the nuclear membrane and nucleus releases ceramide under the action of neurophospholipidase in the nucleus, which can induce apoptosis and other metabolic changes in the nucleus^54^. As a second messenger in the cell, ceramide can promote the phosphorylation of eukaryotic transcription initiation factor 2α, thereby inhibiting protein synthesis. This inhibition is also proportional to the concentration of ceramide^55,56^. Sphingolipid and its metabolites also participate in the pathogen-induced hypersensitivity^57^, host-pathogen interaction^58^, and insulin signal transduction^59^. Therefore, changes of metabolic pathways in the body in response to environmental stress can be effectively evaluated and predicted by understanding how sphingolipid compounds are expressed.

GPI anchoring modification is one of the most common post-translational modifications of eukaryotic cell membrane surface proteins^60^. GPI anchors and GPI-anchored proteins may be involved in a variety of biological and pathological processes, such as recognition and adhesion between cells, transduction of biological signals, bacterial, viral, and parasitic infection, and enzymatic reactions on the cell surface^61^. In the present study, the signaling pathway glycosylphosphatidylinositol (GPI)-anchor biosynthesis was significantly enriched in each salinity group. The expression levels of PE (14:0/16:1(9Z)) were significantly down-regulated as salinity decreased. This finding suggests that GPI played a crucial role in the transduction of biological signals and had a significant effect on cell morphology retention and intercellular adhesion after water loss or absorption by cells during osmotic pressure regulation. The biosynthesis of GPI is initiated from the cytoplasmic side of the endoplasmic reticulum. It was reported that if biosynthesis of GPI is blocked in the first few steps, GPI-anchored proteins may become unstable and the release of the pattern recognition protein alkaline phosphatase of GPI-anchored proteins will change; moreover, change in the activity of alkaline phosphatase can lead to the occurrence of many diseases^62^. In this regard, effective synthesis of GPI is key to maintaining cell structure and function and enhancing intercellular signal transmission when the body responds to environmental changes.

Autophagy is a cell survival mechanism that occurs in response to lack of nutrients and external stress, and it plays a crucial role in maintaining intracellular stability, cell energy metabolism, and cell survival^63^. In this study, the autophagy-other and autophagy-animal signaling pathways were significantly enriched in all salinity groups. The metabolite PE (14:0/16:1(9Z)) in the above pathways were significantly down-regulated. It was presumed that in the response to salinity stress, the proteins and organelles in the cell were degraded by means of autophagy, and these components were reused in the most effective way to maintain cell survival and tissue self-stability. Autophagy has two important functions in cells: first, energy sources in cells can be recycled to accommodate energy requirements; second, cytotoxic proteins and organelles can be scavenged under environmental stress. The environments upon which organisms depend are full of various dynamic balances of coordination and exclusion. In response to various stresses, cells have to adapt to changing metabolic needs and protect internal processes from damage^64^, and autophagy fulfils an important function in promoting cell survival in a response to various kinds of stress. Autophagy also helps organisms resist and regulate inflammation, infection, and immunity^65^. In the present study, the expression levels of *Mn-SOD*, *CAT*, *CTL*, *GP*_*x*_, and *HSP90* increased significantly with the decrease in salinity. This finding suggests that in order to shield the body from oxidative damage due to environmental stress, antioxidant defense mechanisms and immune autophagy were quickly initiated to facilitate scavenging of inflammatory activators (e.g., ROS and inactivated mitochondrial DNA)^66^. In this way, endogenous sources inhibited cellular inflammatory substances from being activated.

To ensure the normal operation of living organisms, lipids must be continuously circulated and reallocated in cells, and autophagy also participates in the recycling and reuse of lipids. This recycling process involves recruitment of lipids from lysosomes via autophagy or endocytosis, which can in turn facilitate degradation of lysosomal hydrolase. The catabolic products resulting from this process, including fatty acids, are reallocated into different cell compartments to support basic cellular functions^67^. As a result, the down-regulation of PE (14:0/16:1(9Z)) as found in this work may be attributed to the degradation to fatty acids by means of autophagy and then reused as an energy source.

In summary, the activities of T-AOC, Na^+^/K^+^-ATP, SOD, and CAT and the content of MDA were significantly higher in S14 than in S22 and S30. The expression levels of *Mn-SOD*, *CAT*, and *CTL* also tended to be up-regulated when the salinity dropped suddenly. These results indicate that the enhanced activity of Na^+^/K^+^-ATPase and related antioxidant enzymes is an important way of maintaining the intracellular and extracellular ion balance as well as general cellular homeostasis during the response of *S. subcrenata* to the sudden drop in salinity. The activity of GP_x_, the content of GSH, and the expression levels of *GP*_*x*,_ and *HSP90* did not increase continuously as the salinity decreased, but the expression levels in S14 were still significantly higher than those in S30. Thus, when the salinity decreased from 30‰ to 22‰ and 14‰, respectively, GP_x_, and CAT together played a dominant role in scavenging ROS and shielding cell structure and function from damage. Metabolomics analysis revealed 361, 271, and 264 metabolites that differed significantly in comparisons of S22 vs. S14, S30 vs. S14, and S30 vs. S22, respectively. Glycerophospholipid metabolism, sphingolipid metabolism, glycosylphosphatidylinositol (GPI)-anchor biosynthesis, autophagy-other, and autophagy-animal pathways were significantly enriched in each salinity group. However, with a decrease in salinity, PC (20:4 (5Z, 8Z, 11Z, 14Z)/P-18: 1 (11Z)), PC (16:0/22: 6 (4Z, 7Z, 10Z, 13Z, 16Z, 19Z)), PE (18:4 (6Z, 9Z, 12Z, 15Z)/24:1 (15Z)), PI (20:1 (11Z)/0:0), phalluside-1, C16 sphinganine, and LacCer (d18:0/14:0) were up-regulated, whereas PI-Cer (d18:1/14:0) and PE (14:0/16:1(9Z)) were down-regulated significantly. Therefore, these nine metabolites can function as metabolic markers, inferring that these metabolites aid in adaptation to sudden drop in salinity. These data provide physiological evidence that changes in salinity affect the metabolism of *S. subcrenata* and could affect their ecology. In addition, this study provides a theoretical basis for selection of areas of appropriate salinity for release of seeds to replenish stocks of *S. subcrenata*.

## Supplementary information

Supplementary information.

## Data Availability

The metabolome datasets supporting the conclusions of this article are available in the [China National GeneBank (CNGB)] repository under the accession number of CNP0000948 [unique persistent identifier and hyperlink to dataset(s) in https://db.cngb.org/search/project/CNP0000948/].
