# Supplementary material for: Effects of a Sudden Drop in Salinity on Scapharca subcrenata Antioxidant Defenses and Metabolism Determined Using LC-MS Non-targeted Metabolomics
Source: Sci Rep. 2020 Apr 30;10:7324. doi: 10.1038/s41598-020-63293-0 (PMC7192903; doi:10.1038/s41598-020-63293-0)
Supplement: Supplementary file 1 — Supplementary information. [file 41598_2020_63293_MOESM1_ESM.pdf]

Effects of a Sudden Drop in Salinity on *Scapharca subcrenata* Antioxidant Defenses and Metabolism Determined Using LC-MS Non-targeted Metabolomics

Zhang Mo[a], Li Li[b], Liu Ying[a,c], Gao Xiaolong[d*]

[a]*Key Laboratory of Experimental Marine Biology, Institute of Oceanology, Chinese Academy of Sciences, Qingdao, 266071, China*

[b]*Marine Biology Institute of Shandong Province, Qingdao, 266104, China*

[c]*Dalian Ocean University, Dalian, 116023, China*

[d]*State Key Laboratory of Marine Environmental Science, College of Ocean and Earth Sciences, Xiamen University, Xiamen, 361102, China.*

***Corresponding author:**

Gao Xiaolong*

College of Ocean & Earth Sciences, Xiamen University, 361102, Xiamen, Fujian Province, China.

Tel.: +86 592 2187420; fax: +86 532 2187420

Email: xlgao@xmu.edu.cn

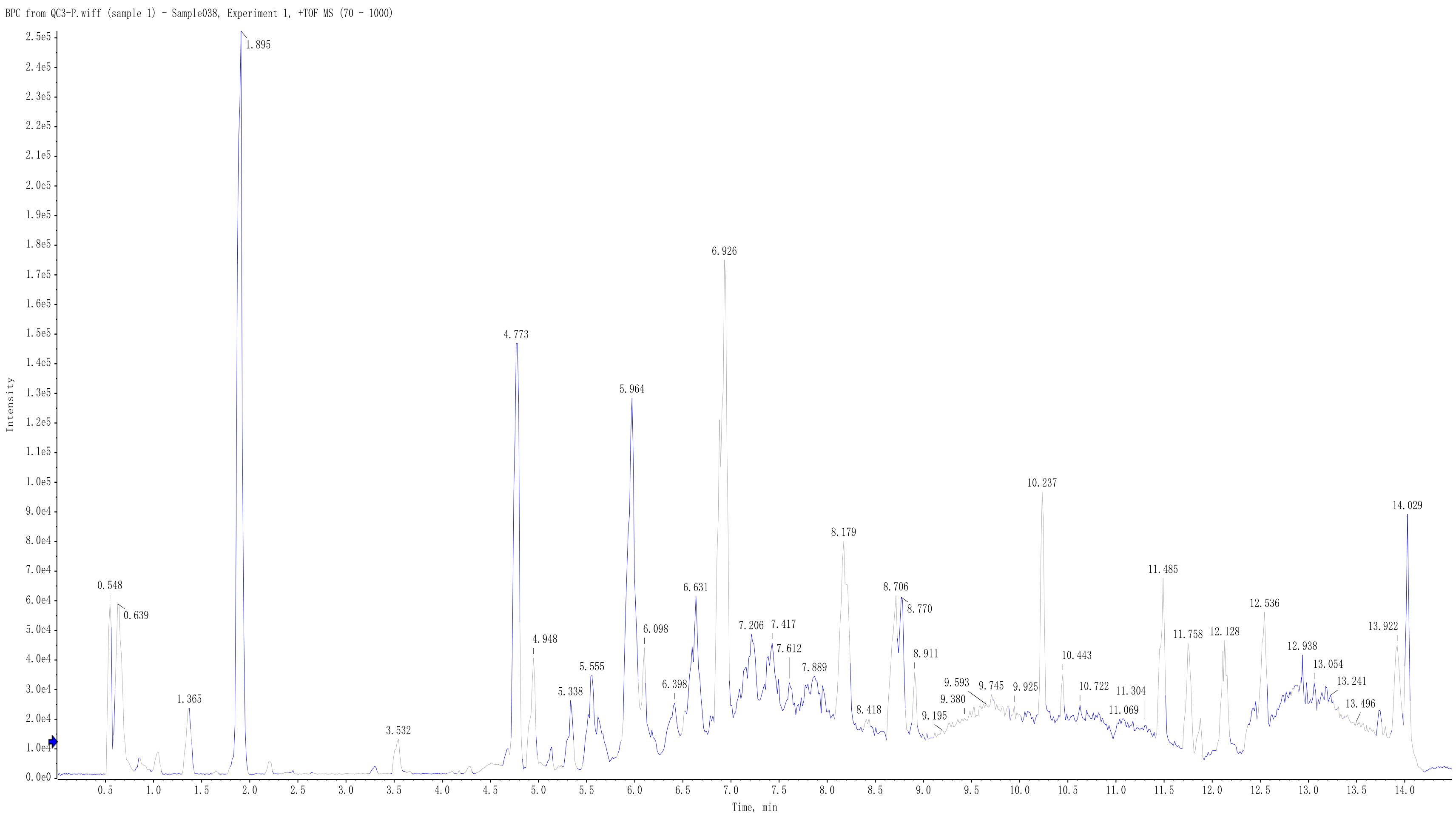
BPC from QC3-P.wiff (sample 1) - Sample038, Experiment 1, +TOF MS (70 - 1000)
Intensity
Time, min
1.895
6.926
4.773
5.964
10.237
14.029
8.179
11.485
6.631
0.548
0.639
8.706
8.770
12.536
7.206
7.417
6.098
4.948
11.758
12.128
13.922
8.911
5.555
7.612
12.938
10.443
7.889
13.054
9.593
9.745
9.925
6.398
10.722
13.241
1.365
5.338
11.304
9.380
13.496
8.418
11.069
3.532
9.195

(a)

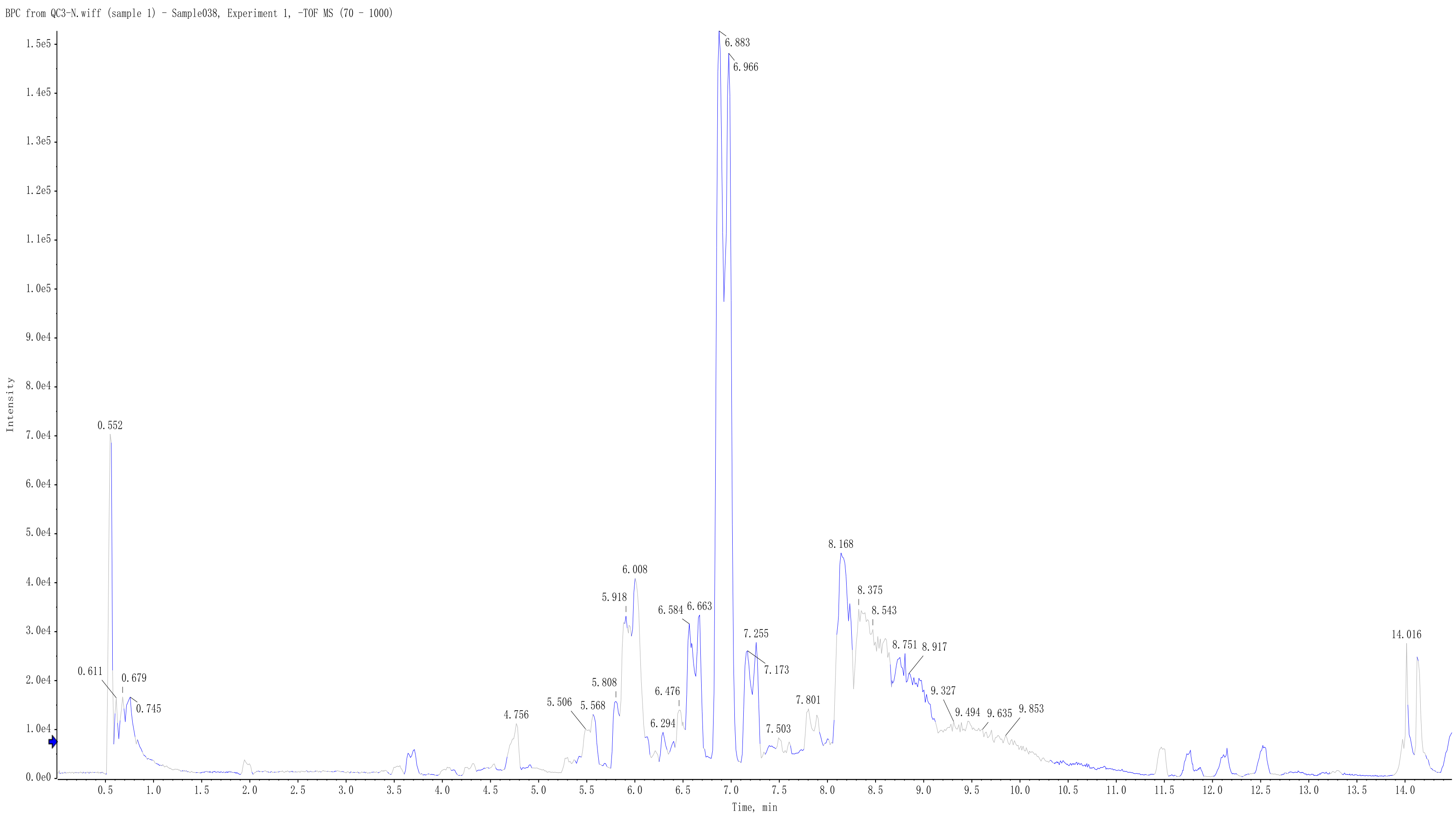

(b)

Supplementary Fig. 1. The base peak chromatogram of positive (a) and negative (b) ion of quality control samples by liquid chromatography-mass spectrometry (LC-MS).

Supplementary Table 1. Significant key metabolites in S22 vs S14, S30 vs 14, and S30 vs S22.

Groups	Metabolites	Ion mode	Metabolic pathway	VIP	*P*-value	log2(FC)	Trend change
	PC(20:4(5Z,8Z,11Z,14Z)/P-18:1(11Z))	+	Glycerophospholipids	31.6145	3.995E-05	1.2671147	↑
	PC(16:0/22:6(4Z,7Z,10Z,13Z,16Z,19Z))	+	Glycerophospholipids	22.1903	0.0001625	1.1560961	↑
	PE(18:4(6Z,9Z,12Z,15Z)/24:1(15Z))	+	Glycerophospholipids	16.9201	9.244E-05	1.1848168	↑
	1-Stearoylglycerophosphoinositol	-	Glycerophospholipids	14.2074	0.0076523	1.0392974	↑
	PI(20:1(11Z)/0:0)	-	Glycerophospholipids	13.1593	0.001027	1.3360517	↑
	PC(18:3(6Z,9Z,12Z)/P-18:1(11Z))	+	Glycerophospholipids	11.7354	0.0001221	0.8921213	↑
	PC(18:1(11Z)/22:6(4Z,7Z,10Z,13Z,16Z,19Z))	+	Unclassified	10.0833	0.0002145	0.8298988	↑
	PON-PE	+	Unclassified	8.40112	0.0004573	-1.4825689	↓
	PE(18:1(11Z)/22:2(13Z,16Z))	+	Glycerophospholipids	8.29901	9.73E-07	1.4967144	↑
	PE(17:1(9Z)/22:4(7Z,10Z,13Z,16Z))	+	Glycerophospholipids	8.00575	0.0091601	0.6036895	↑
	PC(18:2(9Z,12Z)/P-18:1(11Z))	+	Glycerophospholipids	7.61084	1.57E-05	0.7509173	↑
**S22 vs S14**	1,2-Didecanoyl PC	+	Unclassified	7.56056	0.0007681	-1.0984201	↓
	PC(20:5(5Z,8Z,11Z,14Z,17Z)/P-18:1(11Z))	+	Glycerophospholipids	7.52982	0.0001553	0.7743003	↑
	PC(16:0/22:4(7Z,10Z,13Z,16Z))	+	Glycerophospholipids	7.4498	1.257E-05	0.7967662	↑
	PS(18:3(9Z,12Z,15Z)/20:0)	+	Glycerophospholipids	7.43916	0.0052195	-0.8357732	↓
	PS(MonoMe(11,3)/MonoMe(13,5))	+	Glycerophospholipids	7.41211	0.0109954	-0.7319108	↓
	Tryptophanol	+	Indoles and derivatives	7.21497	0.0007861	-0.9413799	↓
	PC(16:0/20:5(5E,8E,11E,14E,17E))[U]	+	Unclassified	7.181	0.0002531	0.7343711	↑
	PS(O-18:0/20:5(5Z,8Z,11Z,14Z,17Z))	+	Glycerophospholipids	7.1164	6.351E-06	0.8598026	↑
	Phalluside-1	+	Sphingolipids	7.04613	6.601E-05	0.5328064	↑
	PEIPC	+	Unclassified	6.58214	0.0164365	-0.8208853	↓
	OHOOA-PC	+	Unclassified	6.48336	0.0007814	-1.4048923	↓
	PE(19:1(9Z)/22:2(13Z,16Z))	+	Glycerophospholipids	6.27696	3.494E-06	1.1928071	↑

PC(O-16:0/16:0)	+	Glycerophospholipids	6.26786	0.0007288	0.7701946	↑
PE(17:0/22:4(7Z,10Z,13Z,16Z))	+	Glycerophospholipids	6.03008	2.618E-05	0.883441	↑
PC(18:4(6Z,9Z,12Z,15Z)/P-18:1(11Z))	+	Glycerophospholipids	5.91573	0.0004694	0.9407182	↑
PC(16:0/0:0)[U] / PC(16:0/0:0)[rac]	+	Unclassified	5.819	0.0119735	-0.617551	↓
C16 Sphinganine	+	Sphingolipids	5.73264	0.0015772	0.7003019	↑
OHODA-PE	+	Unclassified	5.40052	0.0017802	-1.3661103	↓
PS(O-18:0/20:3(8Z,11Z,14Z))	+	Glycerophospholipids	5.39937	2.347E-06	1.0028946	↑
Polyoxyethylene (600) monoricinoleate	+	Fatty Acyls	5.25049	0.0005853	0.8960213	↑
OS-PC	+	Unclassified	5.15051	0.0014491	-1.4990638	↓
PE(22:4(7Z,10Z,13Z,16Z)/P-18:1(11Z))	+	Glycerophospholipids	5.14713	0.0001009	1.0564417	↑
PS(O-16:0/21:0)	+	Glycerophospholipids	5.11633	0.0001006	1.0597072	↑
PE(14:1(9Z)/14:1(9Z))	+	Glycerophospholipids	4.92368	0.0012099	-1.208941	↓
3-Methyl-5-pentyl-2-furanpentadecanoic acid	+	Fatty Acyls	4.89405	0.0063473	-0.4172449	↓
Pubesenolide	+	Steroids and steroid derivatives	4.86353	0.0139115	0.4736187	↑
PE(P-20:0/17:2(9Z,12Z))	+	Glycerophospholipids	4.68349	0.0001175	0.7846142	↑
PS(O-20:0/16:1(9Z))	+	Glycerophospholipids	4.26622	7.016E-05	1.0517226	↑
LysoPC(15:0)	-	Glycerophospholipids	4.22542	0.028102	-0.4808687	↓
3-Epipapyriferic acid	+	Prenol lipids	4.11604	0.0028239	0.5650796	↑
POV-PC	+	Unclassified	4.04274	0.0006052	-1.541069	↓
PE(18:3(6Z,9Z,12Z)/24:1(15Z))	+	Glycerophospholipids	3.87013	0.0002032	1.7583478	↑
PS(14:1(9Z)/20:5(5Z,8Z,11Z,14Z,17Z))	+	Glycerophospholipids	3.75171	0.0011152	1.1071986	↑
PS(14:0/12:0)	+	Glycerophospholipids	3.74384	0.001095	-1.4177544	↓
Squamosinin A	+	Fatty Acyls	3.70974	0.0011192	0.5616766	↑
(23S,25R)-25-hydroxyvitamin D3 26,23-peroxylactone	+	Sterol Lipids	3.69157	0.0017172	0.6800609	↑
PS(P-18:0/20:1(11Z))	+	Glycerophospholipids	3.6632	0.0158104	-0.5763052	↓
D8'-Merulinic acid A	+	Benzene and substituted derivatives	3.65799	0.0028452	0.9538811	↑

	PS(18:1(11Z)/22:2(13Z,16Z))	+	Glycerophospholipids	3.64959	0.0169248	-0.4924594	↓
S30 vs S14	PC(20:4(5Z,8Z,11Z,14Z)/P-18:1(11Z))	+	Glycerophospholipids	25.1206	0.0346892	0.7432462	↑
	PC(16:0/22:6(4Z,7Z,10Z,13Z,16Z,19Z))	+	Glycerophospholipids	23.7851	0.0099915	0.9419651	↑
	PE(18:4(6Z,9Z,12Z,15Z)/24:1(15Z))	+	Glycerophospholipids	16.5398	0.006001	0.789456	↑
	N-stearoyl histidine	-	Fatty Acyls	14.4793	0.0202463	-0.6449837	↓
	PE(17:1(9Z)/22:4(7Z,10Z,13Z,16Z))	+	Glycerophospholipids	9.85944	0.0182447	0.5795272	↑
	PS(O-18:0/20:5(5Z,8Z,11Z,14Z,17Z))	+	Glycerophospholipids	7.80007	0.0025981	0.638249	↑
	PE(18:1(11Z)/22:2(13Z,16Z))	+	Glycerophospholipids	7.75092	0.0051598	1.005999	↑
	PON-PE	+	Unclassified	7.6529	0.03318	-0.6702407	↓
	3-Methyl-5-pentyl-2-furanpentadecanoic acid	+	Fatty Acyls	7.63056	0.0030731	-0.3989846	↓
	PC(18:2(9Z,12Z)/P-18:1(11Z))	+	Glycerophospholipids	7.14972	0.0067833	0.4836584	↑
	PC(16:0/20:5(5E,8E,11E,14E,17E))[U]	+	Unclassified	6.84782	0.0149609	0.4596391	↑
	PC(20:5(5Z,8Z,11Z,14Z,17Z)/P-18:1(11Z))	+	Glycerophospholipids	6.81244	0.0251691	0.4974039	↑
	Tryptophanol	+	Indoles and derivatives	6.71838	0.0317434	-0.464449	↓
	PC(18:4(6Z,9Z,12Z,15Z)/P-18:1(11Z))	+	Glycerophospholipids	6.14761	0.0158799	0.7399752	↑
	Volicitin	+	Unclassified	6.14422	0.0475189	-0.6043486	↓
	PE(17:0/22:4(7Z,10Z,13Z,16Z))	+	Glycerophospholipids	5.8563	0.0049311	0.5853129	↑
	Phalluside-1	+	Sphingolipids	5.7787	0.0060531	0.2185092	↑
	PC(16:0/22:4(7Z,10Z,13Z,16Z))	+	Glycerophospholipids	5.68652	0.042744	0.4269271	↑
	7,7-dimethyl-5,8-Eicosadienoic Acid	-	Unclassified	5.31493	0.003973	-0.2539698	↓
	PE(19:1(9Z)/22:2(13Z,16Z))	+	Glycerophospholipids	5.1869	0.0105898	0.6759367	↑
	PS(O-16:0/21:0)	+	Glycerophospholipids	5.18134	0.0105293	0.794877	↑
	1-(2-methoxy-nonadecanyl)-sn-glycero-3-phosphoethanolamine	+	Unclassified	5.15898	0.0320989	-0.7722234	↓
	OHODA-PE	+	Unclassified	4.96072	0.0454571	-0.6411137	↓
	PS(O-18:0/20:3(8Z,11Z,14Z))	+	Glycerophospholipids	4.82782	0.0038205	0.5921454	↑

PE(22:4(7Z,10Z,13Z,16Z)/P-18:1(11Z))	+	Glycerophospholipids	4.7792	0.021706	0.7301638	↑
PE(14:1(9Z)/14:1(9Z))	+	Glycerophospholipids	4.70568	0.0453615	-0.6343824	↓
N-palmitoyl histidine	-	Fatty Acyls	4.51774	0.0256113	-0.8557081	↓
PS(14:1(9Z)/20:5(5Z,8Z,11Z,14Z,17Z))	+	Glycerophospholipids	4.49668	0.0129756	1.037919	↑
2-O-ethyl PAF C-16	+	Unclassified	4.35384	0.0009497	-1.319407	↓
Prenol	+	Alcohols and polyols	4.09565	0.0263598	-0.667393	↓
PE(18:0/22:6(4Z,7Z,10Z,13Z,16Z,19Z))	-	Glycerophospholipids	3.98448	0.0052406	0.7197946	↑
Verimol B	+	Benzene and substituted derivatives	3.836	0.0454862	1.7606392	↑
(+)-Cloprostenol methyl amide	+	Unclassified	3.74562	0.0270455	-1.1338595	↓
PE(P-20:0/17:2(9Z,12Z))	+	Glycerophospholipids	3.67156	0.0289757	0.3587862	↑
PS(O-18:0/18:3(6Z,9Z,12Z))	+	Glycerophospholipids	3.65133	0.0153623	-1.1950111	↓
POV-PC	+	Unclassified	3.57526	0.0377675	-0.6385929	↓
PE(13:0/18:3(6Z,9Z,12Z))	+	Glycerophospholipids	3.56125	0.0447787	-0.5650025	↓
PC(O-16:0/0:0)	-	Glycerophospholipids	3.48631	0.0196034	-1.2055041	↓
Annoglabasin F	+	Prenol lipids	3.42267	0.0128993	-0.6935751	↓
PC(20:0/P-18:1(11Z))	+	Glycerophospholipids	3.41334	0.0018701	0.38657	↑
Squamosinin A	+	Fatty Acyls	3.37732	0.0365969	0.3548035	↑
Cloversaponin I	+	Prenol lipids	3.35252	0.0226622	-0.7331109	↓
PS(14:0/12:0)	+	Glycerophospholipids	3.31789	0.0422042	-0.6085843	↓
PE(O-18:0/0:0)	+	Glycerophospholipids	3.30541	0.0220918	-0.8013402	↓
Rollinecin A	-	Fatty Acyls	3.30361	0.0127097	0.3029716	↑
PE(17:2(9Z,12Z)/22:4(7Z,10Z,13Z,16Z))	+	Glycerophospholipids	3.2804	0.0021311	0.5316832	↑
N-oleoyl histidine	-	Fatty Acyls	3.28016	0.035828	-0.8462211	↓
Madlongiside C	+	Prenol lipids	3.26513	0.0287882	0.4214612	↑
1-(2-methoxy-eicosanyl)-sn-glycero-3-phosphoserine	-	Unclassified	3.24724	0.0467501	-0.470553	↓
N-stearoyl valine	+	Fatty Acyls	3.1941	0.041796	0.3340449	↑

S30 vs S22	PC(20:4(5Z,8Z,11Z,14Z)/P-18:1(11Z))	+	Glycerophospholipids	27.8551	0.0128336	0.5238686	↑
	PI(20:1(11Z)/0:0)	-	Glycerophospholipids	18.6343	0.0066575	1.0580986	↑
	N-stearoyl histidine	-	Fatty Acyls	15.7842	0.0178618	0.7115652	↑
	PE(18:4(6Z,9Z,12Z,15Z)/24:1(15Z))	+	Glycerophospholipids	13.3157	0.0239541	0.3953608	↑
	PC(18:3(6Z,9Z,12Z)/P-18:1(11Z))	+	Glycerophospholipids	10.6436	0.0170408	0.4285393	↑
	C16 Sphinganine	+	Sphingolipids	8.90962	0.0026271	0.6925864	↑
	PC(18:1(11Z)/22:6(4Z,7Z,10Z,13Z,16Z,19Z))	+	Unclassified	8.65473	0.0259693	0.3528822	↑
	Phalluside-1	+	Sphingolipids	8.20791	0.0014069	0.3142973	↑
	Pubesenolide	+	Steroids and steroid derivatives	8.05657	0.0200994	0.5200591	↑
	Volicitin	+	Unclassified	7.62116	0.0264229	0.7721875	↑
	PON-PE	+	Unclassified	7.06306	0.0294634	-0.8123282	↓
	PC(16:0/22:4(7Z,10Z,13Z,16Z))	+	Glycerophospholipids	6.79436	0.0093721	0.3698391	↑
	PE(18:1(11Z)/22:2(13Z,16Z))	+	Glycerophospholipids	6.7028	0.0181074	0.4907154	↑
	PC(16:0/0:0)[U] / PC(16:0/0:0)[rac]	+	Unclassified	6.41377	0.0339085	-0.4421714	↓
	Tryptophanol	+	Indoles and derivatives	6.35933	0.0212956	-0.476931	↓
	PC(O-16:0/16:0)	+	Glycerophospholipids	6.34577	0.0162327	0.4256355	↑
	OHOOA-PC	+	Unclassified	6.01261	0.0167925	-0.8273367	↓
	PE(19:1(9Z)/22:2(13Z,16Z))	+	Glycerophospholipids	5.88718	0.0073262	0.5168703	↑
	PC(18:2(9Z,12Z)/P-18:1(11Z))	+	Glycerophospholipids	5.7802	0.0396285	0.2672589	↑
	1-(2-methoxy-nonadecanyl)-sn-glycero-3-phosphoethanolamine	+	Unclassified	5.74338	0.0176803	0.8264036	↑
	PC(20:5(5Z,8Z,11Z,14Z,17Z)/P-18:1(11Z))	+	Glycerophospholipids	5.73419	0.0421019	0.2768964	↑
	OS-PC	+	Unclassified	5.62633	0.0029097	-1.0270802	↓
	PE(17:0/22:4(7Z,10Z,13Z,16Z))	+	Glycerophospholipids	5.21937	0.0024963	0.2981281	↑
	PS(O-18:0/20:3(8Z,11Z,14Z))	+	Glycerophospholipids	5.20059	0.0010258	0.4107492	↑

PE(P-20:0/17:2(9Z,12Z))	+	Glycerophospholipids	5.18207	0.006714	0.4258281	↑
CerP(d18:0/16:0)	+	Sphingolipids	5.08374	0.0136934	1.3733662	↑
PE(18:3(6Z,9Z,12Z)/24:1(15Z))	+	Glycerophospholipids	4.93864	0.0005825	1.196884	↑
N-palmitoyl histidine	-	Fatty Acyls	4.89547	0.0343464	0.9417067	↑
OHODA-PE	+	Unclassified	4.67349	0.0219943	-0.7249965	↓
4(5)-EpDPE methyl ester	+	Unclassified	4.6665	0.0122953	-1.1455152	↓
OOV-PG	+	Unclassified	4.34582	0.0054689	1.1412126	↑
(23S,25R)-25-hydroxyvitamin D3 26,23-peroxylactone	+	Sterol Lipids	4.23615	0.0344767	0.4116876	↑
Prenol	+	Alcohols and polyols	4.19083	0.0348281	0.6934012	↑
OHOHA-PE	+	Unclassified	4.02789	0.0054564	-0.9099446	↓
POV-PC	+	Unclassified	3.78993	0.0059696	-0.902476	↓
2-O-ethyl PAF C-16	+	Unclassified	3.78545	0.022927	1.2076716	↑
PC(O-16:0/0:0)	-	Glycerophospholipids	3.68853	0.0259151	1.2799356	↑
PC(18:0/22:4(7Z,10Z,13Z,16Z))	+	Glycerophospholipids	3.51868	0.0033175	0.9238036	↑
PS(14:0/12:0)	+	Glycerophospholipids	3.41814	0.0160537	-0.8091701	↓
8,8a-Deoxyoleandolide	-	Polyketides	3.39174	0.0023888	0.8016663	↑
Cer(d16:2(4E,6E)/20:1(11Z)(2OH))	+	Sphingolipids	3.34799	0.0001115	0.5141754	↑
PS(12:0/14:1(9Z))	+	Glycerophospholipids	3.21105	0.0040211	-1.1593421	↓
18-acetoxy-1alpha,25-dihydroxyvitamin D3	+	Sterol Lipids	3.20877	0.0282174	0.5842656	↑
PG(a-13:0/i-12:0)	+	Glycerophospholipids	3.18772	0.0094486	0.2606497	↑
17a-Hydroxypregnenolone	-	Steroids and steroid derivatives	3.07933	0.020485	0.7628377	↑
hydroxyrepaglinide	+	Piperidines	3.03764	0.0211618	0.9869506	↑
17alpha,20alpha-Dihydroxypregn-4-en-3-one	+	Steroids and steroid derivatives	3.00918	0.0213868	0.6027753	↑
PE(O-18:0/0:0)	+	Glycerophospholipids	2.97024	0.0326288	0.7009272	↑
PC(O-18:1(11Z)/0:0)	+	Glycerophospholipids	2.93022	0.0393885	0.7562051	↑

PC(20:0/P-18:1(11Z))	+	Glycerophospholipids	2.92682	0.0135125	0.2588625	↑

"+", metabolite was detected under positive mode; "–", metabolite was detected under negative mode.
